# Reviewer acknowledgement 2014

**DOI:** 10.1186/1759-8753-5-3

**Published:** 2014-02-25

**Authors:** Nancy L Craig, Thomas H Eickbush, Cédric Feschotte, Henry L Levin

**Affiliations:** 1 Johns Hopkins School of Medicine, 725 N. Wolfe Street, 502 PCTB, Baltimore, MD 21205, USA; 2 Department of Biology, University of Rochester, River Campus, Box 270211, Rochester, NY 14627-0211, USA; 3 Department of Human Genetics, University of Utah School of Medicine, Salt Lake City, UT 84112, USA; 4 Section on Eukaryotic Transposable Elements, Laboratory of Gene Regulation and Development, Eunice Kennedy Shriver National Institute of Child Health and Human Development, National Institutes of Health, Bethesda, MD 20892, USA

## Abstract

**Contributing reviewers:**

The Editors of *Mobile DNA* would like to thank all our reviewers who have contributed to the journal in volume 4 (2013).

## 

Wenfeng An

United States of America

Irina Arkhipova

United States of America

Peter Atkinson

United States of America

Mark Batzer

United States of America

Marlene Belfort

United States of America

Jef Boeke

United States of America

Stephane Boissinot

United States of America

Guillaume Bourque

Canada

Alexandros Bousios

Greece

Julio Camarero

United States of America

Shawn Christensen

United States of America

Edward Chuong

United States of America

Richard Cordaux

France

Joan Curcio

United States of America

Wayne Decatur

United States of America

Prescott Deininger

United States of America

Fred Dyda

United States of America

David Edgell

Canada

José Eirín-López

United States of America

Michael Evgen'ev

Russia

Xiang Gao

United States of America

Valer Gotea

United States of America

Nigel Grindley

United States of America

Fabien Guidez

France

Kyudong Han

United States of America

Dustin Hancks

United States of America

Zsuzsanna Izsvak

Germany

Ning Jiang

United States of America

Patricia Johnson

United States of America

Koichi Kawakami

Japan

Sébastien Leclercq

France

Emmanuelle Lerat

France

Elgion Loreto

Brazil

Francois Michel

France

John Moran

United States of America

Ulrich Muller

United States Minor Outlying Islands

Anja Nagel

Germany

Christian Parisod

Switzerland

Sarah Schaack

United States of America

Gerald Schumann

Germany

Patrick Sinn

United States of America

Jonathan Staley

United States of America

Junji Takeda

Japan

Nicolas Toro

Spain

Zhijian (Jake) Tu

United States of America

Gregory Van Duyne

United States of America

Chantal Vaury

France

David Wood

United States of America

Guojun Yang

Canada

Steven Zimmerly

Canada

